# Treatment of Internal Hemorrhoids by Endoscopic Sclerotherapy with Aluminum Potassium Sulfate and Tannic Acid

**DOI:** 10.1155/2015/517690

**Published:** 2015-07-12

**Authors:** Yuichi Tomiki, Seigo Ono, Jun Aoki, Rina Takahashi, Shun Ishiyama, Kiichi Sugimoto, Yukihiro Yaginuma, Yutaka Kojima, Michitoshi Goto, Atsushi Okuzawa, Kazuhiro Sakamoto

**Affiliations:** Department of Coloproctological Surgery, Faculty of Medicine, Juntendo University, 2-1-1 Hongo, Bunkyo-ku, Tokyo 113-8421, Japan

## Abstract

*Objective*. A new sclerosing agent for hemorrhoids, aluminum potassium sulfate and tannic acid (ALTA), is attracting attention as a curative treatment for internal hemorrhoids without resection. The outcome and safety of ALTA sclerotherapy using an endoscope were investigated in the present study. *Materials and Methods*. Subjects comprised 83 internal hemorrhoid patients (61 males and 22 females). An endoscope was inserted and retroflexed in the rectum, and a 1st-step injection was applied to the upper parts of the hemorrhoids. The retroflexed scope was returned to the normal position, and 2nd–4th-step injections were applied to the middle and lower parts of the hemorrhoids under direct vision. The effects of endoscopic ALTA sclerotherapy were determined by evaluating the condition of the hemorrhoids using an anoscope and interviewing the patient 28 days after the treatment. *Results*. A cure, improvement, and failure were observed in 54 (65.1%), 27 (32.5%), and 2 (2.4%) patients, respectively, treated with ALTA. Complications developed in 4 patients (mild fever in 3 and hematuria in 1). Recurrence occurred in 9.6%. *Conclusions*. The results of the present study suggest that endoscopic ALTA has the potential to become a useful and minimally invasive approach for ALTA sclerotherapy.

## 1. Introduction

Hemorrhoids are a very common anal disease and, when formed on the upper and lower sides of the dentate line, are classified as internal and external hemorrhoids, respectively. Internal hemorrhoids are the most common anal disease, the symptoms of which include hemorrhage and prolapse.

Internal hemorrhoids are resolved in part by conservative treatments with suppositories and ointments, in addition to lifestyle improvements and the avoidance of straining on defecation; however, subsequent treatments may be necessary when symptoms become aggravated and interfere with daily living activities. The treatment of this disease without resection is desirable because internal hemorrhoids are mostly benign.

Several treatment options are available for patients who do not respond to conservative medical management [1–4]. Rubber band ligation and injection sclerotherapy have been the mainstay of nonsurgical treatments for more than a century and are considered to sufficiently treat hemorrhoids. A meta-analysis of 18 randomized trials that compared various treatment methods for hemorrhoids concluded that rubber band ligation was more effective than sclerotherapy and also that patients who underwent ligation were less likely to need subsequent therapy [5]. However, injection sclerotherapy represents a simple and safe palliative treatment for hemorrhoids. The most common sclerosing agent used is 5% phenol almond oil, which is mainly effective for hemorrhage; however, its effects on prolapse are considered to be insufficient [5].

A new sclerosing agent for hemorrhoids, aluminum potassium sulfate and tannic acid (ALTA: Zione; Mitsubishi Pharma Corporation, Osaka, Japan), is effective for not only hemorrhaging from, but also the prolapse of internal hemorrhoids and is expected to replace surgical treatments [6–8].

Injection sclerotherapy with ALTA for hemorrhoids is now performed in Japan, and its efficacy has been reported [6]. ALTA resolves prolapse and hemorrhage after defecation and has several advantages over surgery in that it is associated with fewer complications, such as pain and hemorrhage after treatment, and shortens the treatment period. Therefore, ALTA has been attracting attention as a new hemorrhoid treatment method without resection.

On the other hand, ALTA damages tissue, and complications, such as rectal ulcer and rectal stenosis, have been reported due to the misplacement of injections [9]. Thus, it has been recommended at workshops that ALTA injections be performed after acquiring sufficient knowledge of anal diseases and fully understanding the surgical procedure. Injection sclerotherapy with ALTA is performed through a procedure termed “4-step injections,” which differs from that of conventional injection sclerotherapy in that the agent is injected into 4 regions of the hemorrhoids (upper, deep middle, shallow middle, and lower parts) using an injection needle exclusive for ALTA. Since ALTA is mainly injected into the submucosa, we considered that endoscopic injections using a colonoscope are applicable to ensure submucosal injections [10]. Furthermore, since the injection needle for ALTA is too long to be used for injections into the submucosal layer, an endoscopic injection needle may assist in preventing misplacement of the injection [11].

In the present study, we investigated the outcomes of endoscopic ALTA sclerotherapy and its safety with a literature review.

## 2. Materials and Methods

### 2.1. Patients

Subjects comprised 83 patients with internal hemorrhoids (61 males and 22 females) who required treatment for hemorrhage or prolapse between January 2009 and December 2011 and were unsatisfied with conservative steroid suppository treatments.

ALTA was only indicated for internal hemorrhoids, and acute, thrombosed, strangulated, and external hemorrhoids were excluded from the indication. Concomitant resection of anal polyps and skin tags was not performed.

Since the endoscopic administration of ALTA was not included among the procedures recommended at workshops, this study was performed following approval by the Ethics Committee of our hospital (approval number 488) and obtaining informed consent.

### 2.2. Technique of Endoscopic ALTA Sclerotherapy

**Figure 1:**
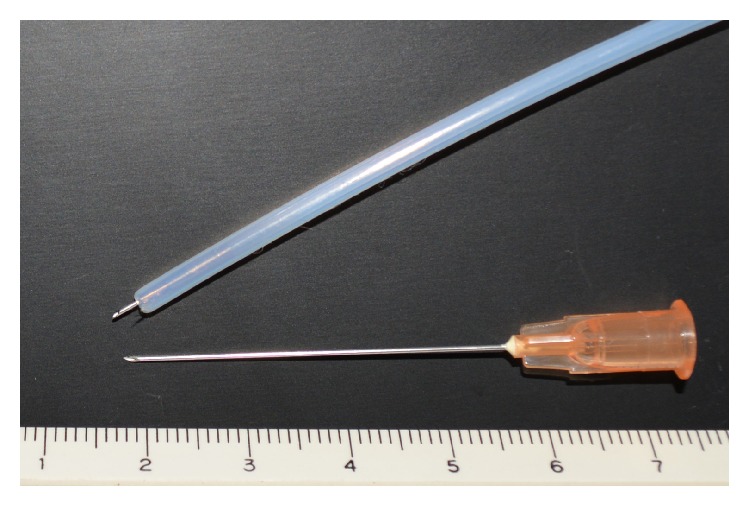
Injection needle. Upper: endoscopic injection needle (25G blunt needle with a length of 3 mm, Top Corporation). Lower: injection needle exclusive for ALTA (25G blunt needle with a length of 38 mm, Nipro Corporation).

Endoscopic ALTA sclerotherapy was performed on all patients at the outpatient clinic (day procedure), and a pretreatment was applied following that for colonoscopy. A thin endoscope was used, and its tip was covered with a transparent hood. A blunt needle (25G, 3 mm in length, Top Corporation, Tokyo, Japan) was used as the endoscopic injection needle (Figure 1).

The treatment was conducted in the left lateral decubitus position after attaching an electrocardiograph and oxygen monitor, and no anesthesia was applied around the anus.

**Figure 2:**
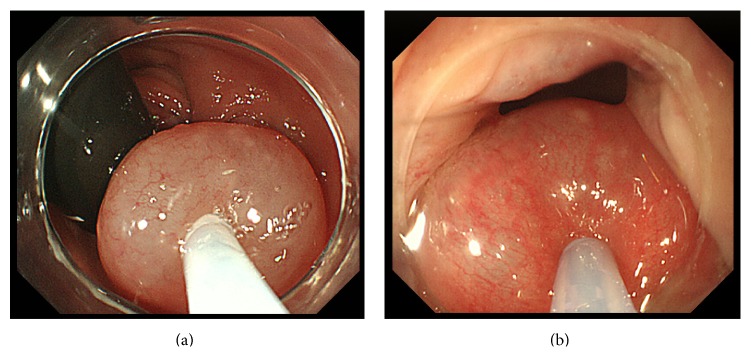
(a) ALTA injection with retroflection of the endoscope as the first step. (b) ALTA injection with a forward-viewing direction as the second to fourth steps.

An endoscope was inserted and retroflexed in the rectum. After observations, the 1st-step injection was applied to the upper pole of the hemorrhoids (Figure 2(a)).

The 1st-step injection was applied to 2-3 sites (3 mL/injection) while keeping the endoscope retroflexed. The retroflexed scope was then returned to the normal position, and the 2nd–4th-step injections (2-3 mL/injection) were applied under direct vision (Figure 2(b)). When the endoscope could not be retroflexed in the rectum, the 1st-step injection was applied under direct vision. Injections were applied while confirming that ALTA had been injected into the submucosa on the monitor. Injections were immediately discontinued when the patient complained of pain [10]. The mean dose of the agent per patient was 14.4 ± 4.0 mL.

The scope was removed after the injections. The local region was then massaged with the index finger to diffuse ALTA throughout the region in order to avoid retention at the injection site, and the procedure was completed [6].

### 2.3. Investigation Items

Patient backgrounds (age, gender, Goligher's classification, ALTA injection dose, and adverse events) and outcomes (treatment effects and recurrence) were investigated.

The effects of ALTA sclerotherapy were determined by evaluating the condition of the hemorrhoids using an anoscope and interviewing the patient 28 days or more after the treatment based on the following evaluation criteria: When the internal hemorrhoid size had decreased on anoscopy and prolapse and hemorrhage after defecation had resolved, the treatment was judged to be markedly effective (cure). When the internal hemorrhoid size had decreased on anoscopy and prolapse and hemorrhage after defecation had remitted but not completely, the treatment was judged to be effective (improvement). When symptoms remained the same after ALTA sclerotherapy regardless of the extent of internal hemorrhoid size reductions, the treatment was determined to be ineffective (failure). During the follow-up, 52 patients who went to the hospital as outpatients were interviewed and examined, while 31 patients who did not go to hospital were interviewed by the surgeon over the phone one year after ALTA sclerotherapy. The follow-up period ranged from 10 to 62 months with a median of 27 months.

### 2.4. Safety of Endoscopic ALTA Sclerotherapy

**Figure 3:**
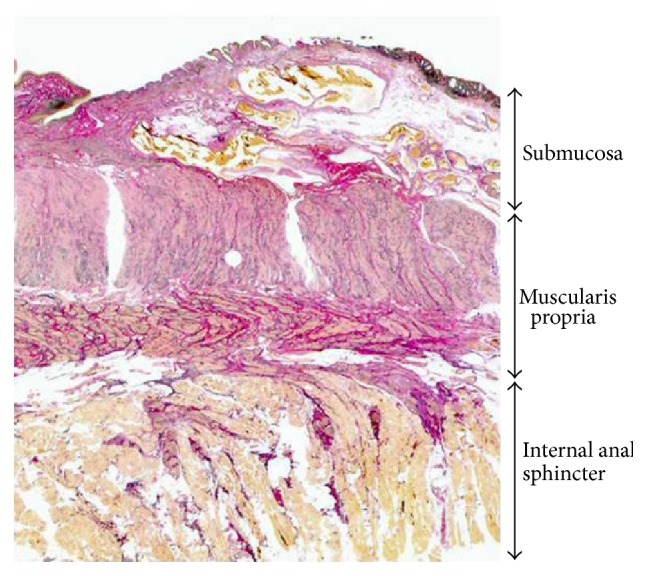
Distance between the mucosa and muscular layer in the lower rectum.

In ALTA sclerotherapy, the agent is mainly injected into the submucosa; however, misplacement of the injection into the muscular layer may occur. In order to verify the safety of endoscopic ALTA sclerotherapy, normal anal regions without tumor infiltration were collected from specimens resected from 12 rectal cancer patients who underwent abdominoperineal resection at our department and were subjected to Elastica van Gieson staining. The distance between the mucosa and muscular layer was measured at 1-2 cm on the oral side from the dentate line, the location at which the rectal venous plexus causing internal hemorrhoids is present, in order to investigate the safety of the endoscopic injections (Figure 3).

### 2.5. Comparison with Conventional Injection Sclerotherapy

Conventional injection sclerotherapy with 5% phenol almond oil (sclerosing agent) was compared with ALTA using previous findings [12–15]. Furthermore, conventional ALTA sclerotherapy with an anoscope was similarly examined based on a literature review [6, 7, 16–19].

## 3. Results

### 3.1. Background of Endoscopic ALTA Sclerotherapy

ALTA was endoscopically injected into 61 male and 22 female patients (mean age: 60.4 years). The most common Goligher's classification grade of the internal hemorrhoids was III, accounting for 81.9% (68 patients), followed by grade II in 12 patients (14.5%). Three grade IV cases (3.6%) were not complicated by thrombosed external hemorrhoids. ALTA sclerotherapy was conducted to ameliorate prolapse.

No symptoms, such as bradycardia, blood pressure reductions, or discomfort, developed during the procedure. Complications developed in 4 patients (4.8%): fever between 37 and 38°C was observed in 3 patients, and hematuria developed 17 days after the procedure in other patients but was remitted by conservative treatment (Table 1).

### 3.2. Therapeutic Effects of Endoscopic ALTA

Among the patients treated with endoscopic ALTA, a cure, improvement, and failure were observed in 54 (65.1%), 27 (32.5%), and 2 (2.4%) patients, respectively, treated with ALTA. Goligher's classification of the 2 failure cases' grade was IV. ALTA was not injected into external hemorrhoids. The two failed cases showed soiling of the underwear after the ALTA injection and no improvements were observed in discomfort or itching. Efficacy was noted in 97.6% when cured and improved cases were combined, suggesting that the short-term outcome of ALTA sclerotherapy was favorable (Table 2). ALTA was fast-acting, with its effects being observed immediately after the injections had been performed in some patients.

Recurrence occurred in 8 patients (9.6%), and hemorrhoids recurred within one year in 4. In the fastest case, prolapse was noted after 91 days.

Endoscopic ALTA sclerotherapy was repeated in 6 out of the 8 recurrence cases. In the other 2 cases, one patient requested surgery and the other continued suppository treatment.

Recurrence was not detected in 5 out of the 6 patients retreated with endoscopic ALTA; however, one patient was not satisfied with the effects and underwent surgery at another institution 3 months after second ALTA sclerotherapy.

### 3.3. Distance between the Mucosa and Muscular Layer in the Lower Rectum

In the 12 rectal cancer patients (8 males and 4 females, mean age: 64.3 years) who underwent abdominoperineal resection, the distance between the mucosa and muscular layer at 1-2 cm on the oral side from the dentate line was 3.3 ± 0.4 mm.

### 3.4. Comparison with Conventional Injection Sclerotherapy

The short-term outcomes of ALTA appeared to be superior to those of 5% phenol in many studies. However, previous findings suggested that ALTA was more effective than 5% phenol. Anal pain was previously reported to be the main complication of these agents [6, 13, 14]. Previous studies also indicated that recurrence was more common and additional therapy was required more frequently with ALTA than with 5% phenol. However, a 4-year follow-up study by Yano et al. showed no significant difference in effectiveness or the need for additional therapy between ALTA and 5% phenol.

The results of the present study, which were obtained with a flexible endoscope, suggested that no significant differences existed between 5% phenol and ALTA sclerotherapy with an anoscope (Table 3).

## 4. Discussion

Hemorrhoids are the most common anal disease. Hemorrhoidectomy is often performed as a surgical treatment for internal hemorrhoids but is associated with postoperative pain, longer recovery times, and complications such as bleeding and anal stricture. Therefore, less invasive treatments are desired for the treatment of this disease without resection. Pile suture by the Farag method has traditionally been employed as a nonexcisional method for hemorrhoids [20]. Various treatment methods, such as procedure for prolapse and hemorrhoids (PPH) and transanal hemorrhoidal dearterialization (THD), have recently been developed and employed [21, 22]. Rubber band ligation and injection sclerotherapy have traditionally been employed as effective treatments and have been the mainstay of nonsurgical treatments for more than a century. Previous studies reported that injection sclerotherapy was less effective than rubber band ligation at controlling symptoms and achieving long-term outcomes [4, 5, 15]. Furthermore, the recurrence rate was lower with rubber band ligation than with injection sclerotherapy. Since the advent of aluminum potassium sulfate and tannic acid (ALTA), ALTA sclerotherapy has been employed as a major nonexcisional method in Japan.

ALTA was prepared by partially changing the additives of Xiaozhiling, a sclerosing agent that was approved for injection sclerotherapy for internal hemorrhoids in 2005 in China. The active ingredient of ALTA, aluminum potassium sulfate, induces aseptic acute inflammation after being injected into hemorrhoids. Fibrosis of the hemorrhoid occurs in the repair process and the hemorrhoid scleroses and recesses. Vascular constriction occurs immediately after the injection, which reduces blood flow to the hemorrhoid and stops hemorrhage. On the other hand, tannic acid inhibits excess acute inflammation induced by aluminum potassium sulfate, thereby reducing secondary tissue damage [23]. ALTA is only indicated for internal hemorrhoids; thus, acute, strangulated, and external hemorrhoids are excluded from the indication.

ALTA is injected using a procedure termed “4-step injections.” Anesthesia may be applied around the anus (preinjection anesthesia). After insertion of the anoscope, ALTA is injected into the submucosa of internal hemorrhoids. At workshops, practitioners have been instructed to shake the needle tip to confirm that it has not been inserted into the muscular layer when its contact with the muscular layer is felt and then inject ALTA. The injection of ALTA into the narrow submucosa by operating an anoscope with one hand and adjusting a light with the other hand requires skill; therefore, we considered application of the endoscopic mucosal resection technique using an endoscopic injection needle to be safer for submucosal injections. Since this is not instructed at workshops, we carefully performed this procedure by applying a pretreatment following that for colonoscopy to prevent accidents, covering the endoscope tip with a transparent hood to add an anoscopic function and ensuring that ALTA was injected into the submucosa by observing a bulge in the injected regions on a monitor.

The distance between the mucosa and muscular layer was previously reported to be 3.3 ± 0.4 mm in resected rectal cancer preparations [24]. Therefore, the possibility of misplacing injections into the muscular layer using an endoscopic injection needle with a length of 3 mm is low unless the needle is strongly pressed into hemorrhoids. Inversely, an injection into a shallow submucosal region with the endoscopic injection needle is also a matter of concern, and it may be important to carefully massage the treated region after injections in order to sufficiently diffuse the drug solution.

The effectiveness of endoscopic injections of sclerosing agents into internal hemorrhoids has already been reported [25, 26], and the principle of endoscopic ALTA sclerotherapy is the same as that of conventional ALTA sclerotherapy, excluding the use of a flexible endoscope.

Endoscopic ALTA sclerotherapy does not require anesthesia, the visual field is favorable, the operative field may be shared through a monitor, and deep insertion of the needle is prevented. Furthermore, the screening step of colorectoanal neoplasia is not necessary. However, this procedure requires a pretreatment with laxatives and the cost of endoscopy cannot be calculated.

In order to determine the therapeutic effects of endoscopic ALTA, its clinical outcomes were compared with those of conventional anoscopic ALTA sclerotherapy. A combination of cured and improved cases showed that endoscopic ALTA remitted symptoms in 97.6%. The incidence of complications was 4.8%; this value was slightly higher when mild symptoms, such as discomfort of the anus, were included and appeared to be slightly lower than that caused by conventional ALTA sclerotherapy, suggesting that this is a less invasive and more effective method than conventional ALTA sclerotherapy. Hematuria developed 17 days after ALTA injections in one patient. Urological complications have been suggested to result from an anteriorly misplaced injection into the prostate, urethra, or periprostatic venous plexus [11]. Even though the endoscopic injection needle was used, injections may have been deep; therefore, injections need to be closely observed in order to ensure submucosal injections on the monitor, and the immediate discontinuation of injections is necessary when the patient complains of pain. In addition, it is important to sufficiently massage the treated region to prevent the local retention of injected ALTA, thereby reducing the risk of complications.

Recurrence occurred in 9.6% of patients. The standard dose of ALTA was 9–13 mL per hemorrhoid and 27–39 mL for 3 hemorrhoids. The endoscopic dose of ALTA applied was low (14 mL per patient), which may have been the cause of recurrence [6]. We set the dose at a lower level as a cautionary measure because endoscopic ALTA is different to the recommended procedure; however, elevations in the dose while paying attention to complications may be necessary. In the present study, sufficient therapeutic effects were achieved for grade II and III internal hemorrhoids at the low dose. A higher ALTA dosage and subsequent treatments, including surgery, may be needed for grade IV internal hemorrhoids. Since ALTA sclerotherapy is not a radical treatment for hemorrhoids and it is positioned between conservative suppository treatments and surgery, a recurrence rate of approximately 10% may be acceptable.

Regarding the long-term outcomes of ALTA, a symptom-free rate of 53% after 4 years has been reported [19]; however, since ALTA is also known to be effective for recurrence, endoscopic ALTA sclerotherapy, which is applicable as a day procedure, may be used as the first-line treatment for recurrent cases.

The present study had the following limitations: a conventional ALTA method with an anoscope and a randomized prospective trial were not conducted. Therefore, further investigations are warranted.

## 5. Conclusions

Subsequent treatments, including surgery, have been recommended for many cases of internal hemorrhoids for which conservative suppository treatments were ineffective. The results of the present study suggest that the therapeutic effects of ALTA were similar to those of surgery and also that it has the potential to become a breakthrough treatment method for curing internal hemorrhoids without resection. Therefore, endoscopic ALTA has the potential to become a minimally invasive and useful approach for ALTA sclerotherapy.

## Figures and Tables

**Table 1:** Patient characteristics.

	Endoscopic ALTA (*n* = 83)
Sex (male : female)	61 : 22
Age	60.4 ± 14.0
Grade of hemorrhoids	
Goligher's classification	
I	0 (0.0%)
II	12 (14.5%)
III	68 (81.9%)
IV	3 (3.6%)
Injection dose (mL)	14.4 ± 4.0
Complications	4 (4.8%)

**Table 2:** Short-term outcomes of ALTA sclerotherapy.

	Effectiveness of ALTA			
	Cure	Improvement	Failure	Recurrence
Endoscopic ALTA (*n* = 83)	54 (65.1%)	27 (32.5%)	2 (2.4%)	8 (9.6%)

**Table 3:** Comparison with conventional injection sclerotherapy.

References	Patients (*n*)	Follow-up (months)	Sclerosing agents	Effectiveness (%)	Complications (%)	Recurrence (%)	Additional therapy (%)
Greca et al. 1981 [27]	43	12	5% phenol	70.0	0	30.2	14.0
Gartell et al. 1985 [12]	109	33	5% phenol	70.0	0	—	25.7
Walker et al. 1990 [13]	28	48	5% phenol	35.7	70.2	64.2	14.3
Santos et al. 1993 [14]	189	48	5% phenol	60.3	0.5	8.5	22.2
Kanellos et al. 2003 [15]	80	48	5% phenol	17.5	36.3	63.0	30.0
Shi 1997 [16]	21,361	36	Xiaozhiling	99.0	4.6	1.0	—
Takano et al. 2006 [6]	95	12	ALTA	94.0	49.0	16.0	—
Tokunaga et al. 2010 [17]	784	4–30	ALTA	96.0	1.8	—	—
Hachiro et al. 2011 [7]	448	29	ALTA	—	3.1	—	3.1
Miyamoto et al. 2012 [18]	28	8–21	ALTA	100	21.4	10.7	—
Yano et al. 2014 [19]	57	48	ALTA	53	—	—	22.8
Present study 2015	83	27	ALTA	97.6	4.8	9.6	8.4

—: results absent or not clearly reported in the text.
